# From AI dependence to reflective collaboration: psychological ownership, competency anxiety, and perceived support in AI-assisted learning

**DOI:** 10.3389/fpsyg.2026.1903784

**Published:** 2026-07-15

**Authors:** Xiaoling Li, Xujin Xian, Peitao Du

**Affiliations:** 1 The Catholic University of Korea, Bucheon, Republic of Korea; 2 Chongqing Second Normal University, Chongqing, China; 3 Al-Farabi Kazakh National University, Almaty, Kazakhstan

**Keywords:** AI-assisted learning, cognitive offloading, competency anxiety, generative artificial intelligence, human-AI collaboration, perceived educational support, psychological ownership, self-regulated learning

## Abstract

Generative artificial intelligence (GenAI) can improve efficiency in academic work. However, it also raises a central psychological question: when AI helps produce a task, why do some learners still experience the final work as their own, whereas others experience it as polished but psychologically distant? This study examines psychological ownership and competency anxiety in AI-assisted learning, distinguishing between dependent AI outsourcing and reflective human–AI collaboration. An exploratory qualitative interview-based design with supplementary background information was used. The background form was used only for sample description and interview preparation, followed by in-depth interviews with 50 undergraduate and postgraduate students from five Chinese universities, interviews with 10 faculty members and administrators, and 128 student critical incident records. Data were analyzed through a hybrid deductive–inductive thematic analysis, with NVivo 14 used as a supporting tool for code management and matrix comparison. In participants’ accounts, dependent AI outsourcing was associated with weaker psychological ownership, described in terms of reduced cognitive and authorial presence during planning, reasoning, and revision. This weakened sense of ownership was, in turn, linked to shallow memory, difficulty explaining an individual’s work, reduced meaning-making, and stronger competency anxiety. Reflective human–AI collaboration, by contrast, was associated with retained ownership when students planned before using GenAI, evaluated AI output, revised it in their own voice, and could explain their final decisions. Perceived educational support, including feedback, clear AI-use guidance, and psychologically safe learning environments, was described as helping students treat AI use as a learnable practice rather than a hidden shortcut. Faculty and administrators also framed these patterns as issues of assessment feasibility, workload, policy clarity, and curriculum design, rather than solely as student choice. Because the data were collected at five Chinese universities, the proposed model is presented as context-sensitive: the observed dynamics may be amplified where assessment is strongly product-oriented, formative feedback is constrained, and AI use is difficult to discuss openly. This study contributes to psychological ownership theory by extending it to human–AI interaction and proposing that, in this setting, ownership depends on cognitive and authorial presence rather than solely on task completion.

## 1 Introduction

Generative artificial intelligence (GenAI) has rapidly become embedded in university learning, writing, coding, feedback seeking, and assessment preparation (Bearman et al., 2023; Kasneci et al., 2023; Yan et al., 2024). The central psychological issue is, therefore, not only whether students use GenAI but also how they experience work that has been partly produced through human–AI interaction. Similar AI-assisted products may reflect very different learning processes. One student may submit a polished assignment but be unable to explain its reasoning, whereas another may use GenAI to question an argument, improve language, or compare alternatives while retaining control over the final decisions. Recent student-perception studies similarly show that GenAI use varies across tasks, disciplines, and policy environments (Baek et al., 2024; Johnston et al., 2024; Kelly et al., 2023; Stöhr et al., 2024). To examine these differences, this study distinguishes two ideal-typical patterns of use. Dependent AI outsourcing refers to using GenAI to generate the main structure, reasoning, or language of academic work before the learner has formed sufficient personal understanding. Reflective human–AI collaboration involves using GenAI to provide targeted support after the learner has formed an initial intention, question, argument, or plan, while the learner remains responsible for evaluation and revision. These two patterns are not treated as fixed groups of students, but as sensitizing contrasts on a continuum: the same learner may use GenAI dependently in one task and reflectively in another. The distinction helps focus the analysis on whether learner agency, cognitive control, and authorial responsibility are preserved during the learning process.

Psychological ownership provides the central lens for this analysis. It refers to whether students experience completed work as genuinely their own (Pierce et al., 2001; Pierce et al., 2003). In the GenAI context, ownership is examined through students’ ability to remember and explain their work, perceive it as meaningful, and judge whether AI-supported performance aligns with their personally owned competence. Existing discussions have generated important insights into acceptance, academic integrity, institutional policy, and assessment redesign (Bittle and El-Gayar, 2025; Cotton et al., 2024; Miao and Holmes, 2023; Moorhouse et al., 2023; Perkins, 2023). Less is known about students’ lived psychological experience after completing AI-assisted work: why some work feels personally owned, memorable, and meaningful, whereas other work feels distant, difficult to explain, and associated with anxiety about their real competence.

Two research questions guide the analysis. RQ1 asks how students experience psychological ownership, explainability, meaning-making, and competency anxiety under dependent AI outsourcing and reflective human–AI collaboration. RQ2 asks how assessment climate, feedback practices, AI-policy clarity, and perceived educational support shape these AI-use patterns as psychological boundary conditions. This qualitative framing aligns with reporting standards for interview-based and qualitative research (Levitt et al., 2018; Tong et al., 2007).

**Figure 1:**
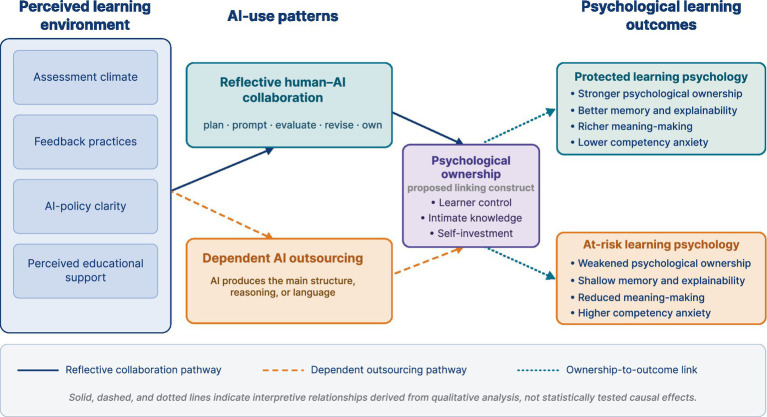
Conceptual map of psychological ownership and contextual support in GenAI-assisted learning. This is a theory-generating conceptual map derived from qualitative thematic analysis; all connections are interpretive associations grounded in participants’ accounts, not statistically tested or causal effects. The three line styles distinguish three types of interpretive relationship rather than different causal strengths: the reflective human–AI collaboration pathway (solid), the dependent AI outsourcing pathway (dashed), and the ownership-to-outcome link (dotted). Psychological ownership is shown as the central construct linking AI-use patterns to psychological learning outcomes and is interpreted through three pathways drawn from the literature: learner control, intimate knowledge of the work, and self-investment. It is presented as a proposed linking construct to be examined in future research, not as a statistically established mediator. This should, therefore, be read as a conceptual map, not as a tested mediation or path model.

The study makes three conceptual contributions. First, it extends psychological ownership theory to GenAI-assisted learning by showing that ownership can depend on cognitive and authorial presence rather than solely on unaided production. Second, it conceptualizes competency anxiety as a possible metacognitive signal of a discrepancy between AI-supported performance and personally owned capability. Third, it links reflective human–AI collaboration with self-regulated learning by identifying planning, evaluation, revision, and explanation as practices that help preserve ownership. Figure 1 presents these relationships as a theory-generating conceptual map rather than as a statistically tested model.

## 2 Literature review

### 2.1 GenAI-assisted learning beyond acceptance and integrity

Research on GenAI in higher education has expanded rapidly from acceptance and usefulness (Davis, 1989; Venkatesh et al., 2003) to AI literacy, academic integrity, institutional policies, and student/faculty perceptions (Almassaad et al., 2024; Baek et al., 2024; Bearman et al., 2023; Kim et al., 2025; Perkins, 2023; Von Garrel and Mayer, 2023). These topics are necessary, but they do not fully explain how students experience learning when GenAI becomes embedded in everyday academic tasks. A student may accept GenAI as useful while still losing confidence in their own ability. A university may introduce integrity rules while still failing to help students learn how to collaborate with AI reflectively. This study, therefore, treats GenAI-assisted learning as a psychological process rather than only a technological or disciplinary issue.

GenAI can support learning by helping students ask better questions, compare alternative explanations, receive timely feedback, or revise unclear writing (Farrokhnia et al., 2024; Kasneci et al., 2023; Mai et al., 2024; Yan et al., 2024). It may also weaken learning when it is used to produce a complete answer before the learner has attempted to understand the task (Tlili et al., 2023). The same tool can, therefore, support or undermine learning, depending on its role in the process. This is why the distinction between dependent AI outsourcing and reflective human–AI collaboration is central to this study.

### 2.2 Dependent AI outsourcing, cognitive offloading, and shallow learning

Cognitive offloading occurs when people rely on external tools to reduce mental effort (Risko and Gilbert, 2016). Offloading is not inherently negative. Students have always used books, calculators, search engines, notes, and peers. The educational risk lies in offloading the operations that constitute learning itself: formulating a problem, organizing evidence, making judgments, revising language, and explaining choices. When GenAI completes these operations too early, students may receive an answer without building the internal structure needed to recall, explain, or defend it (Grinschgl et al., 2021; Sparrow et al., 2011).

Dependent AI outsourcing is a specific form of academic work offloading. It occurs when the student allows GenAI to decide the main direction of the task, produce the initial structure, formulate the argument, or write the core language. The student may then edit the output superficially, but the system has already made the major intellectual moves. Such use can improve short-term efficiency while weakening the student’s relationship to the product, a concern also raised in recent debates on GenAI, assessment, and academic integrity (Bittle and El-Gayar, 2025; Gerlich, 2025; Lim et al., 2023). The assignment may be submitted successfully, but the learning process has been bypassed (Kofinas et al., 2025).

This pattern is particularly important in writing, coding, literature review, and conceptual analysis, where generative systems are increasingly used for educational and research-related tasks (Kasneci et al., 2023; Lee et al., 2024; Rahman and Watanobe, 2023; Yan et al., 2024). These tasks are not only products to be graded but also learning processes through which students practice reasoning, judgment, expression, and explanation. If GenAI performs the process before students engage with it, the result may look academically competent while the learner remains psychologically absent (Zawacki-Richter et al., 2019).

### 2.3 Psychological ownership, memory, and meaning-making

Psychological ownership refers to the feeling that an object, idea, product, or piece of work is psychologically “mine” (Pierce et al., 2001; Pierce et al., 2003). In educational contexts, psychological ownership is built through effort, agency, self-investment, and responsibility (Asatryan et al., 2013). Students are more likely to own their work when they have chosen the question, struggled with evidence, made revisions, and defended the final argument. They are less likely to own work that is produced fully formed by an external system.

According to psychological ownership theory, ownership emerges through three main pathways: perceived control over the object or task, intimate knowledge of it, and self-investment of time, effort, or identity (Avey et al., 2009; Pierce et al., 2001; Pierce et al., 2003). In GenAI-assisted learning, dependent outsourcing may undermine all three. The student does not fully control the reasoning process, does not develop intimate knowledge of the content, and invests relatively little personal effort or identity in the final product. Reflective collaboration, by contrast, may preserve these pathways when the learner sets the goal, evaluates AI output, revises it in the learner’s own voice, and remains responsible for the final work.

Psychological ownership helps connect cognitive and affective dimensions of GenAI-assisted learning. When students cannot recall what they submitted, cannot explain why a claim was made, or cannot defend a coding decision, the issue is not simply poor memory (Grinschgl et al., 2021; Risko and Gilbert, 2016; Sparrow et al., 2011). It also indicates a weakened relationship between the learner and the work. The task no longer carries the student’s own decision-making history. This helps explain why some students report emptiness after AI-assisted assignments: the final product may be acceptable, but it does not feel personally meaningful or authorially grounded.

Meaning-making is the deeper layer of this problem. Academic work is not only a display of correct answers but also a way for students to connect knowledge with personal understanding, disciplinary identity, and future competence (Pintrich, 2004; Zimmerman, 2002). When GenAI is used reflectively, it can enrich meaning-making by offering examples, counterarguments, and alternative perspectives. When it is used dependently, it can remove the struggle through which meaning is often formed (Panadero, 2017).

### 2.4 Reflective human–AI collaboration and self-regulated learning

Reflective human–AI collaboration is grounded in self-regulated learning. Self-regulated learners plan their work, monitor progress, evaluate feedback, and revise strategies (Panadero, 2017; Pintrich, 2004; Zimmerman, 2002). In the GenAI context, these processes can be described as a cycle: plan, prompt, evaluate, revise, and own. Students first clarify what they want to do, then ask GenAI for a specific kind of help, evaluate the response, revise it in relation to their own intention, and finally take responsibility for the result. This cycle corresponds to the forethought, performance, and self-reflection phases of self-regulated learning. Table 1 summarizes how this reflective collaboration cycle aligns with the major phases of self-regulated learning.

**Table 1:** Alignment of self-regulated learning phases and reflective GenAI collaboration.

SRL phase	GenAI collaboration behavior
Forethought	Setting goals, clarifying one’s own question, and planning what to ask AI
Performance	Prompting, monitoring AI output, and checking accuracy, relevance, and logic
Self-reflection	Revising in one’s own voice, explaining decisions, and taking ownership of the final work

This cycle does not require students to avoid AI. On the contrary, reflective collaboration may involve frequent AI use. The difference is that AI does not replace the learner’s judgment. Reflective users may ask AI to challenge an argument, identify weaknesses in a draft, summarize opposing views, translate technical language, or polish grammar. However, they remain responsible for deciding whether the output is accurate, relevant, ethical, and consistent with their own voice (Carless and Boud, 2018; Tai et al., 2018). Thus, reflective collaboration protects psychological ownership by keeping students engaged in the learning process (Nicol and Macfarlane-Dick, 2006).

### 2.5 Learning environment: assessment climate, feedback practices, AI-policy clarity, and perceived educational support

Learning environments shape students’ AI-use patterns. Assessment climate refers to what students believe a course rewards: final products, originality, process evidence, oral explanation, reflection, or improvement. Feedback practices refer to whether students receive information that helps them monitor and revise their work (Boud and Molloy, 2013; Hattie and Timperley, 2007; Nicol and Macfarlane-Dick, 2006). AI-policy clarity refers to whether students understand which kinds of GenAI use are acceptable, discouraged, or require disclosure. In this study, perceived educational support refers to students’ subjective perceptions that teachers, departments, and university systems provide guidance, AI-literacy resources, advising, psychological safety, and practical support for responsible GenAI use. The concept is informed by organizational support theory but is reframed here through educational, teacher, academic, and institutional perspectives on learning support.

These contextual factors influence the practical logic of AI use. When a course values only the final output, outsourcing becomes attractive because the process remains invisible. When a course requires staged submission, draft comparison, oral defense, and reflective explanation, outsourcing becomes less useful because students must still understand and own the work (Carless and Boud, 2018). Clear AI policies also matter. If students do not know what is allowed, they may hide their use and receive no guidance (Kofinas et al., 2025; Miao and Holmes, 2023; Moorhouse et al., 2023). If responsible use is explained and supported, students can learn how to collaborate with GenAI without surrendering agency (Michel-Villarreal et al., 2023).

Perceived educational support is especially important because students often experience AI use as morally and academically ambiguous (Chan and Hu, 2023). Recent studies across several national contexts show that students’ GenAI use and concerns are shaped by policy clarity, disciplinary expectations, access, and confidence in responsible use (Almassaad et al., 2024; Kim et al., 2025; Stöhr et al., 2024; Von Garrel and Mayer, 2023). Students may know that AI is useful but fear being judged as lazy or dishonest. Supportive teachers, departments, and university-level services can change this climate by treating GenAI literacy as part of academic development rather than merely a compliance problem. This support-based framing is informed by organizational support theory (Eisenberger et al., 1986; Rhoades and Eisenberger, 2002).

### 2.6 Task affordance for outsourcing as a boundary condition

Task type matters not simply because disciplines differ, but because tasks differ in how readily GenAI can produce an acceptable final product while leaving the learner’s reasoning process unobserved. We use the task outsourcing affordance to describe this property. Outsourcing affordance is higher when the output is easily generated, process evidence is not required, authorship is difficult to trace, and students are not asked to explain or defend their decisions. It is lower when work is iterative, embodied, publicly discussed, or accompanied by visible drafts, demonstrations, or oral defense.

This distinction avoids treating humanities, STEM, or arts disciplines as inherently more or less vulnerable. The same discipline can contain both high- and low-affordance tasks. For example, a final essay graded only as a product may permit extensive outsourcing, whereas a seminar paper developed through staged discussion and oral defense may not. Similarly, a coding task may be highly outsourceable when only functioning output is assessed, but less so when students must diagnose errors and explain each design choice. Task outsourcing affordance is, therefore, treated as a boundary condition on the ownership process rather than as a core stage within it.

### 2.7 Competency anxiety in the AI era

Competency anxiety is defined here as a future-oriented, domain-specific concern that an individual’s own academic or professional capabilities may be eroding, becoming insufficient, or failing to develop because AI-supported performance exceeds what the individual can independently produce. Its core appraisal is a perceived discrepancy between visible performance and personally owned capability. This makes it different from ordinary academic stress about a particular grade or deadline and from AI-use or technology anxiety about operating the tool itself (Markus and Nurius, 1986; Schneider and Preckel, 2017).

The construct should also be distinguished from its possible regulatory consequences. Competency anxiety itself is the future-oriented concern; adaptive recalibration, shame, concealment, or avoidance are downstream responses rather than parts of the definition. The same concern may prompt a student to rebuild independent skills when the discrepancy feels specific, controllable, and discussable, but it may produce shame or hidden dependence when rules are ambiguous, support is weak, and the concern is interpreted as evidence of global personal inadequacy. In this sense, competency anxiety can sometimes function as a metacognitive warning signal, but that function is conditional rather than definitional. A student may also have high AI-operation self-efficacy while experiencing high competency anxiety about domain-specific reasoning, writing, coding, or problem-solving skills (Bong and Skaalvik, 2003; Chan and Hu, 2023; Compeau and Higgins, 1995; Crompton and Burke, 2023). In this study, competency anxiety is characterized interpretively from participants’ accounts rather than being treated as an already validated scale.

## 3 Methods

### 3.1 Research design

This study employed an exploratory qualitative interview-based design with supplementary background information (Levitt et al., 2018; Tong et al., 2007). A brief background information form was administered first to record minimal categorical contextual information: age range, sex, academic level, broad discipline, frequency of GenAI use, main AI-supported task types, and willingness to discuss a recent AI-assisted learning episode. These items were used only for sample description and interview preparation; they were not treated as scale scores, covariates, or variables for cross-case statistical comparison. The qualitative analysis comprised two connected strands: a learner-experience strand based on student interviews and critical incident records, and a stakeholder-context strand based on faculty and administrator interviews. The strands were first examined separately and then compared for convergence, divergence, and contextual interpretation (Palinkas et al., 2015).

This design was appropriate because the research questions concern students’ lived experiences and the meaning they attach to GenAI-assisted academic work. Background information alone cannot explain why a student feels that an AI-assisted essay is not “mine,” why another student uses AI while retaining authorship, or how assessment and feedback make direct outsourcing seem practically reasonable. Qualitative data allow these mechanisms to be examined in students’ own language (Braun and Clarke, 2006; Hsieh and Shannon, 2005), while the background information helps contextualize the interview sample and tailor follow-up questions (Levitt et al., 2018).

The analysis was informed by sensitizing expectations rather than statistically testable hypotheses. Dependent AI outsourcing was expected to be associated with weaker psychological ownership, reduced explainability, and lower meaning-making, whereas reflective human–AI collaboration was expected to be linked to stronger ownership and lower competency anxiety, particularly when students perceived stronger educational support. The affordance of task outsourcing and the national, institutional, and disciplinary context were examined as possible boundary conditions rather than assumed to be causal variables. The resulting model is, therefore, positioned as a theoretical concept mapping: it organizes qualitative relationships that future longitudinal, experimental, or mixed-method research can test rather than claiming causal verification in this study (Eisenhardt, 1989; Fereday and Muir-Cochrane, 2006; Nowell et al., 2017).

### 3.2 Participants and sampling

Fifty undergraduate and postgraduate students were recruited from five Chinese universities. Following purposive and maximum-variation sampling, the sites were selected to span varied institutional contexts, with 10 students from each site (Palinkas et al., 2015). These contextual differences were relevant because prior work suggests that institutional stratification may be associated with class size, feedback capacity, assessment design, and support resources, that is, with the kinds of learning-environment conditions this study examines. The purpose was to widen the range of learning contexts sampled, not to rank or statistically compare institution types. Maximum-variation sampling also spanned different academic levels, disciplines, and AI-use patterns. The final student sample comprised 28 female and 22 male participants aged 20–25 years; 36 were undergraduates, and 14 were postgraduates; 22 were from STEM fields, 19 from humanities and social sciences, and 9 from arts-related fields. This distribution supported maximum variation but did not create statistically balanced disciplinary subgroups; therefore, discipline-related patterns are interpreted cautiously as task-affordance patterns rather than as representative disciplinary comparisons. De-identified participant profiles are provided in Supplementary Table S2 (students) and Supplementary Table S3 (faculty and administrators) (Campbell et al., 2013).

Ten faculty members or academic administrators were also interviewed. They included professors, associate professors, lecturers, and an academic affairs administrator across a broad range of experience, from 0–5 years to 16+ years. Their interviews were not treated merely as background corroboration. They formed a distinct stakeholder strand examining how teachers and administrators defined problematic dependence, interpreted ownership and explainability, assessed the feasibility of process-oriented assessment, and understood policy and support constraints. This strand was subsequently compared with student accounts to identify both convergence and stakeholder-specific tensions (Fereday and Muir-Cochrane, 2006; Hsieh and Shannon, 2005).

### 3.3 Data collection

After ethical approval had been obtained, data collection proceeded in four phases. First, students completed a brief background information form recording their age range, sex, academic level, broad discipline, frequency of GenAI use, main AI-supported task types, and willingness to discuss a recent AI-assisted learning episode (Supplementary Table S5). The form did not collect scale scores, psychological outcome variables, or covariates for statistical modeling; it only supported sample contextualization and interview tailoring. Second, 50 student interviews were conducted. Most interviews lasted approximately 60–120 min (median, approximately 90 min), with the length varying according to the amount of detail participants provided about concrete AI-use episodes. All interviews were conducted by the research team across the five sites over approximately 3 months, audio-recorded with participants’ consent, and transcribed verbatim, yielding approximately 95 h of recorded interview data. This distribution of fieldwork across the team and sites made the volume of data manageable within the data-collection window. All interviews were conducted in Chinese by members of the research team who are native Mandarin speakers. The research team includes native Mandarin-speaking doctoral researchers with bilingual proficiency in English and experience publishing in international journals. Excerpts selected for reporting were first translated into English by one bilingual team member and then independently checked against the original Chinese transcripts by a second bilingual researcher to preserve meaning and nuance. Any discrepancies in translation were resolved through discussion. The original Chinese wording was retained throughout the analysis phase. Before final reporting, all quoted interview excerpts and critical incident record IDs were checked against the de-identified transcript and CIR logs to confirm code-excerpt consistency (Supplementary Table S8). Students were asked what they used GenAI for, what they did before and after using it, whether they could remember and explain the final work, whether the work felt like their own, and how the learning environment influenced their choices; the full student and faculty/administrator interview protocols are provided in Supplementary Methods 2 and 3. Third, 10 faculty and administrator interviews were conducted, each lasting approximately 45–60 min. These interviews explored observed changes in student work, assessment practices, feedback constraints, AI-policy implementation, and support needs. Fourth, students were invited to submit 2-3 critical incident records within 1 week after the interview using a structured template (Supplementary Table S6). In total, 128 critical incident records were collected, providing brief written accounts of specific AI-use episodes that complemented and were analyzed alongside the interview data. The data-collection procedure was reported in line with qualitative interview standards (Levitt et al., 2018; Tong et al., 2007).

### 3.4 Data analysis and trustworthiness

Student interviews, faculty and administrator interviews, and critical incident records were analyzed using a hybrid deductive–inductive thematic analysis of a codebook. This study followed principles of thematic analysis in psychology by identifying patterned meanings across participants’ accounts (Braun and Clarke, 2006), while using a codebook that allowed both theory-driven and data-driven codes (Fereday and Muir-Cochrane, 2006; Hsieh and Shannon, 2005). Student interviews and critical incident records were analyzed as the primary strand of the learner experience. Faculty and administrator interviews were analyzed as a distinct stakeholder strand before cross-strand comparison. This structure allowed faculty data to illuminate institutional feasibility, policy translation, and professional judgment rather than merely confirming student reports.

The analysis proceeded in five stages. First, student interview transcripts and critical incident records were read repeatedly to develop familiarity with learner experiences. Second, an initial coding framework was developed from the research questions and relevant theoretical concepts, including psychological ownership, memory and explainability, meaning-making, self-regulated AI use, dependent AI outsourcing, assessment climate, feedback climate, AI-policy clarity, perceived educational support, competency anxiety, and task outsourcing affordance (Supplementary Table S1). Third, the faculty and administrator interviews were coded separately for definitions of problematic AI dependence, markers of student ownership, assessment and workload constraints, policy ambiguity, curriculum responsibility, and feasible support strategies. Fourth, open coding across both strands was used to identify additional data-driven codes and refine the codebook. Fifth, the full dataset was coded in NVivo 14, after which strand-specific matrices were compared to identify convergence, divergence, and contextual mechanisms across participants, tasks, disciplines, institution types, and data sources (Nowell et al., 2017). A summary of the faculty/administrator analytic strand is presented in Supplementary Table S9.

To enhance trustworthiness, two researchers independently coded an initial subset of student and faculty/administrator transcripts. Specifically, 10 student interview transcripts (20% of the 50 student interviews) and 3 faculty/administrator interview transcripts (30% of the 10 faculty/administrator interviews) were randomly selected for double coding (Campbell et al., 2013; O’Connor and Joffe, 2020). Coding differences were discussed until consensus was reached, and the codebook was refined accordingly. An inter-coder reliability check was conducted on this double-coded subset (Cohen’s kappa = 0.82 for student interviews and 0.79 for faculty/administrator interviews) (Cohen, 1960). Because the analysis followed a structured codebook approach rather than a reflexive thematic analysis design, the reliability check was used as a measure of dependability rather than as a substitute for interpretive analysis. Coding logs, strand-specific matrices, analytical memos, and reflexive notes were maintained throughout the analysis, and the trustworthiness measures are summarized in Supplementary Table S4. Member checking was conducted with selected students and faculty participants. The final integration focused on both student psychological experience and the distinctive institutional judgments, constraints, and support priorities articulated by faculty and administrators.

Theme saturation and reflexivity were also considered during analysis. Reflexive notes were maintained throughout coding to record assumptions, coding decisions, and possible interpretive bias (Braun and Clarke, 2021; Nowell et al., 2017). As part of this reflexivity, the research team examined its own positionality. The team included doctoral researchers and scholars in education and related fields who were familiar with Chinese higher-education settings and had experience using GenAI tools in academic work. The team was attentive to the risk that a prior commitment to the distinction between dependent outsourcing and reflective collaboration could lead to confirmation of expected patterns. It used negative-case analysis, peer debriefing, and member checking to counter this risk. After approximately 45 student interviews, no substantially new first-order codes were identified; the final five interviews and the remaining critical incident records mainly confirmed and refined existing themes (Guest et al., 2020). Because this study was theoretically oriented toward self-regulated learning and reflective human–AI collaboration, the researchers paid particular attention to negative and boundary cases, including students who used GenAI frequently but retained strong ownership, and students who used AI reflectively but still reported competency anxiety. Coding disagreements were resolved by returning to concrete evidence of learner agency, such as whether the student had formed an initial intention, evaluated AI output, revised in their own voice, and could later explain the work. These negative and boundary cases informed the boundary conditions described in the results.

### 3.5 Ethical considerations

This study was reviewed and approved through the ethics review process of the Asia-Pacific Think Tank Research Institute on January 5, 2026, with the review result indicating that the project was approved to proceed as planned. The review classified the project as a minimal-risk social-science and educational research study based on background information collection, semi-structured interviews, and student critical incident records. The project did not involve live animal experiments, clinical trials, biomedical intervention, collection of human biological specimens, or deception involving more than minimal risk. The ethics, consent, anonymization, and data-protection arrangements are summarized in Supplementary Methods 1. Before participation, all participants received information about the study purpose, procedures, data use, privacy protection measures, voluntary participation, and their right to withdraw at any time, consistent with qualitative research reporting expectations (Levitt et al., 2018; Tong et al., 2007). Participants provided written informed consent before participation and could pause the interview, decline to answer any question, or withdraw without penalty. No individual AI-use behavior was reported to teachers or administrators for disciplinary purposes. Names of persons, universities, courses, and other identifying details were removed from interview transcripts, background information forms, and critical incident records. Data were stored in de-identified form and used only for academic research.

## 4 Results

The hybrid deductive–inductive codebook thematic analysis identified seven themes, followed by a brief boundary-case analysis. The results follow the central logic of the study: how learning environments and task affordances shape AI-use patterns; how these patterns are connected with psychological learning outcomes; and how faculty and administrator perspectives complicate or extend student accounts. Table 2 summarizes the themes, key findings, and analyst-inferred psychological processes before the detailed analysis.

**Table 2:** Summary of themes and key findings.

Theme	Theme name	Related question	Key finding	Interpretive psychological process (analyst-inferred)
1	Dependent AI outsourcing and loss of psychological ownership	RQ1	Students using dependent AI outsourcing reported shallow recall, blurred authorship, emotional emptiness, and reduced agency	Reduced control; disrupted self-investment; participant-reported shallow recall
2	Assessment climate and feedback deprivation	RQ2	Outcome-only grading and limited feedback made direct AI outsourcing more rational	External performance pressure; weakened metacognitive monitoring
3	Task affordance, disciplinary context, and institutional boundary conditions	Exploratory	Outsourcing was most visible when AI-generatable outputs, low process visibility, final-product grading, and limited feedback converged	Task outsourcing affordance; contextual vulnerability
4	Reflective human–AI collaboration and self-regulated learning	RQ1/RQ2	Reflective users planned, evaluated, revised, and owned AI-assisted work	Preserved agency; metacognitive monitoring; authorial control
5	Competency anxiety and perceived educational support	RQ1/RQ2	Students described a future-oriented performance–capability discrepancy; support shaped whether anxiety prompted recalibration or shame and concealment	Threatened possible self; conditional metacognitive signal; domain-confidence concern
6	Faculty and administrator perspectives on ownership, feasibility, and policy	RQ2/stakeholder strand	Faculty defined problematic use through explainability and responsibility, while emphasizing workload, policy, and curriculum constraints	Professional judgment; institutional feasibility; stakeholder divergence
7	Psychological ownership–contextual support model for reflective use	Discussion-linked	Visible process requirements and perceived support may help students shift from dependent outsourcing toward reflective collaboration	Contextual support for ownership, self-regulation, and explainability

### 4.1 Dependent AI outsourcing and loss of psychological ownership

The most immediate pattern in the data was the gap between product quality and psychological ownership. Many students using dependent AI outsourcing described AI-assisted work as efficient but psychologically distant. They could submit a coherent essay, report, or code explanation, but after submission, they could not clearly recall the argument or explain why certain decisions had been made. The work was formally theirs, but psychologically, it did not feel theirs fully. This sense of psychological distance reflects disruption of the ownership pathways of control, intimate knowledge, and self-investment: students did not generate the reasoning, did not deeply know the content, and could not locate personal effort in the final product.

One humanities student explained: “Using AI to write that essay? It was fast—really fast. But after finishing, I do not know. What thinking did I actually do? It was like the thinking process just never happened.” (D01). Another student said: “The assignment has my name on it, but the structure and wording were given by AI. I only accepted and modified it” (A05). These statements show that the problem was not merely technical dependence. Students were describing a weakened sense of authorship.

Dependent AI outsourcing also affected memory and explainability. Several students reported that they could not answer follow-up questions about AI-assisted work. A humanities student recalled that when a teacher asked about an argument a week after submission, “I could not explain it, even though I had submitted the essay” (D03). This pattern suggests that GenAI can create a separation between submitted output and internal learning when the student does not participate in the reasoning process.

Another student described the inability to explain their own AI-assisted work: “I submitted a really good essay—my teacher said it was ‘well-structured.’ But when my friend asked me what my main argument was, I just said, ‘I do not know, it’s in there somewhere.’ That’s when I realized I had not actually written it” (C02).

Critical incident records further supported psychological distance. One undergraduate student wrote: “I used AI to write the entire discussion section of my lab report. When the TA asked me a simple question about it in the next class, I could not answer at all. I realized I had just copied and pasted without understanding” (CIR-023). Another record stated: “The AI-generated code passed all test cases, but in the viva I could not explain why a particular loop was there. I felt like a fraud” (CIR-047).

This theme supports RQ1 by showing that dependent AI outsourcing can simultaneously weaken psychological ownership, memory and explainability, and meaning-making. The students were not simply saying that AI made assignments easier. They said this ease came with a loss of involvement. Psychologically, the final product had form, but the student’s own cognitive history was thin; the assignment lacked autobiographical markers of personal effort, choice, and struggle. Similar patterns were also observed in the critical incident records. In records where GenAI was used to generate the main reasoning or structure, participants described difficulty reconstructing the argument or explaining the logic shortly after submission. By contrast, records describing planning, checking, or revising before or after AI use illustrated how students retained a sense of ownership and recalled key decisions when they remained involved in the process.

### 4.2 Assessment climate and feedback deprivation as contextual drivers

The second theme explains why dependent AI outsourcing became attractive. Many students described learning environments in which only the final product mattered. Assignments were often essays, reports, summaries, or programming outputs submitted at the end of a course. Drafts, oral explanation, process notes, and formative feedback were uncommon. In this context, students understood direct AI outsourcing as a strategically efficient response to assessment design.

A student in a final-product-oriented course stated: “Nearly every course requires a final essay. The teacher does not look at drafts and gives only a final score. If only results matter, using AI is the smartest choice” (D06). Another student compared their own effort with a roommate’s AI use: “I spent 3 weeks writing by myself; my roommate used AI and finished in 2 hours. We got the same grade” (D04). Such accounts show how assessment climate can change the perceived value of personal effort.

A student contrasting courses with and without feedback noted: “In the course where the teacher gave comments on our drafts, I did not dare to just copy AI. But in the other course with only a final grade, everyone used AI directly—why would not you?” (D02).

Faculty interviews confirmed that the problem was partly structural. Several teachers wanted to provide more feedback but described large classes, limited time, and heavy administrative workloads. A lecturer teaching a large class said: “I want to give formative feedback, but with 80 students in a class, it is very difficult” (T08). In contrast, teachers who used process-oriented assessment described staged assignments, oral presentations, AI-use declarations, and reflective logs. These practices did not eliminate AI use; they changed its form by making the process and explanation visible.

This theme answers RQ2 by showing that assessment climate and feedback practices can either invite outsourcing or support reflective collaboration. When the process is invisible, outsourcing is efficient. When the process is assessed, students must remain engaged in the work. In psychological terms, feedback and process requirements strengthened metacognitive monitoring by requiring students to explain how they arrived at an answer and why they accepted or rejected AI suggestions.

### 4.3 Task affordance, disciplinary context, and institutional boundary conditions

Across the data, task outsourcing affordance was more informative than discipline alone. High-affordance tasks allowed GenAI to produce a plausible final product while keeping planning, reasoning, revision, and authorship largely invisible. Dependent outsourcing was most visible when four conditions converged: the output was readily generatable, only the final product was graded, follow-up explanation was unlikely, and formative feedback was limited.

Writing-heavy humanities and social-science assignments often displayed this configuration because standardized essays could appear complete without preserving personal interpretation or authorial history. However, this was not an inherent property of those disciplines. Seminar papers developed through staged discussion, source justification, or oral defense had lower outsourcing affordance. The same variation appeared in STEM: code that functioned or produced a correct solution could be outsourced when only the output was assessed, whereas debugging, code walkthroughs, and explanations of design choices kept reasoning visible. Arts and design tasks often had lower affordance because iterative production and visible authorship were built into the task, although ideation and stylistic decisions could still be displaced when AI-use expectations were unclear.

These patterns are, therefore, interpreted as task- and assessment-level boundary conditions rather than statistical subgroup differences. Institution and discipline mattered insofar as they shaped process visibility, feedback capacity, and the requirement to explain decisions. This interpretation is summarized in Supplementary Table S7 and is incorporated into the future-testable propositions.

### 4.4 Reflective human–AI collaboration and self-regulated learning

Reflective users did not avoid GenAI. Many used it frequently. Their difference was that they used it after forming an initial understanding, before finalizing work they still regarded as their own. They described AI as a debate partner, a feedback source, a search assistant, or a polishing tool. They also described moments of evaluation: checking whether AI output was accurate, whether it matched their argument, and whether it sounded like their own voice.

One philosophy student explained: “I write my own argument first. Then I tell AI, like, ‘oppose me.’ If it finds something weak, I revise it. It’s not writing for me—it’s challenging me.” (A03). A design student said: “I write the rough idea first. AI can help me polish or find references, but if I start from AI output, the final work does not feel like mine” (A09). These accounts illustrate the reflective cycle: plan, prompt, evaluate, revise, and own.

A STEM student working on an algorithmic task explained his reflective routine: “I write the algorithm myself first, even if it’s buggy. Then I ask AI: ‘What’s wrong with this?’ I only accept suggestions I understand. If I do not understand the fix, I do not use it. That way, the code is still my logic” (C04).

Critical incident records also captured reflective practices. One student wrote: “I first wrote my own bullet-point outline for the essay. Then I asked AI to suggest three counterarguments. I rejected one because it did not fit my evidence, and rewrote the other two in my own words. The final essay still felt like mine” (CIR-076). Another record noted: “Before using AI to polish my code comments, I made sure I could explain each function line by line. AI just helped me phrase it more clearly—it did not think for me” (CIR-091).

Across the interviews, dependent outsourcing and reflective collaboration appeared as two recurring patterns rather than fixed statistical groups. The classifications are used heuristically to contrast psychological experiences: whether students retained control, understanding, self-investment, and explainability while GenAI was involved.

### 4.5 Competency anxiety and perceived educational support

The fifth theme concerns students’ future-oriented anxiety. Many students did not simply worry about grades or plagiarism. They worried that GenAI might make them appear competent while their real abilities declined. A marketing student asked: “If AI can write reports, what ability am I really developing? What will a company need me for?” (E07). A postgraduate STEM student said: “I am afraid of becoming someone who only checks AI output, not someone who can solve problems” (B03).

Another student described this anxiety in relation to future performance: “I use AI every day. I’m efficient now. But I honestly do not know if I could pass a coding interview without it. That thought keeps me up sometimes” (B04).

Competency anxiety also emerged in critical incident records. A postgraduate student wrote: “I got an A on the AI-assisted literature review, but I knew I could not write anything close to that under exam conditions. That scared me more than a low grade would have” (CIR-112). Another record stated: “I feel like I’m becoming an AI supervisor, not a learner. Even when I check AI outputs carefully, I worry: if the internet went down, could I still solve this problem? Probably not” (CIR-128).

Taken together, these accounts show that competency anxiety was especially salient when students experienced a discrepancy between academic performance and personally owned capability. Students could complete tasks faster, but the speed did not necessarily produce confidence. Analytically, competency anxiety was coded as the future-oriented concern itself: the worry that an individual’s independently available capability was less developed than the submitted performance suggested. This concern was closely connected with psychological ownership because students were less anxious when they could reconstruct, explain, and defend their decisions, even when AI had contributed substantially.

The behavioral response to competency anxiety was coded separately. In some accounts, the concern prompted adaptive recalibration: students reduced direct outsourcing, wrote outlines before prompting, practiced without AI, or checked outputs more carefully. In other accounts, it produced shame, concealment, or continued dependence. The difference appeared to depend on whether the capability gap felt specific and controllable and whether students had clear rules, constructive feedback, and psychologically safe opportunities to discuss and revise their AI use. Competency anxiety can, therefore, operate as a metacognitive warning signal, but it does not do so automatically.

Perceived educational support shaped how competency anxiety was interpreted and managed. Students who received clear guidance, feedback, and non-punitive discussion about GenAI were more likely to describe AI use as a learnable practice. Students who experienced unclear rules or a lack of guidance from teachers were more likely to hide their use and to interpret dependence as a private failure. Faculty participants also recognized this need. One teacher argued that students must learn “how to judge AI, not only how to use AI” (T01). Another noted that curriculum design had not yet caught up with the competencies required in an AI-rich environment (T10).

### 4.6 Faculty and administrator perspectives: ownership, feasibility, and policy

Analyzed as a distinct stakeholder strand, faculty and administrator accounts contributed more than corroboration. First, they tended to define problematic AI dependence through students’ inability to explain, justify, or take responsibility for a product, rather than solely on the frequency of AI use. This perspective aligned with the student distinction between dependent outsourcing and reflective collaboration and shifted attention from detection of tool use to evidence of evaluative judgment. As one teacher put it, students needed to learn “how to judge AI, not only how to use AI” (T01).

Second, faculty located ownership problems within assessment feasibility. Teachers described process-oriented assessment, draft feedback, and oral explanation as desirable but difficult to sustain in large classes and under heavy workloads. A lecturer teaching a large class explained, “I want to give formative feedback, but with 80 students in a class, it is very difficult” (T08). This introduces an institutional tension absent from student accounts: practices that preserve ownership may be pedagogically persuasive yet operationally costly.

Third, administrators and senior faculty emphasized the coordination of curriculum and policy. They viewed isolated prohibitions as insufficient when students lacked shared expectations about disclosure, evaluation, and the capabilities that should remain independently demonstrable; one administrator noted that curriculum design had not yet caught up with the competencies required in an AI-rich environment (T10). A stakeholder divergence therefore emerged. Students often experienced silence or ambiguity as judgment, indifference, or lack of support, whereas faculty described the same silence as a consequence of unclear policy, limited training, and implementation capacity. This divergence helps explain why unclear institutional responses can intensify concealment even when teachers do not intend to create a punitive climate. Supplementary Table S9 summarizes this analytic strand.

### 4.7 Boundary and negative cases

The boundary cases also showed that high AI-use frequency was not equivalent to weak ownership. One postgraduate STEM participant who used GenAI very frequently described a highly reflective pattern: he first wrote his own code structure, then used GenAI to compare solutions, diagnose errors, and check alternative explanations. Because he could still explain the logic and justify which suggestions were accepted or rejected, frequent AI use did not weaken ownership. This case suggests that learner control, intimate knowledge, and self-investment, rather than frequency of AI use alone, are the critical psychological conditions.

One high-frequency AI user who retained strong ownership explained: “I use AI for almost every assignment, but I always start with my own plan. I treat AI as a smart junior colleague—I tell it what to do, I check its work, and I overrule it when I disagree. I can explain every line of the final product. That’s why it still feels mine” (B07).

A second boundary pattern involved students who used AI reflectively but still reported competency anxiety. For example, one postgraduate STEM participant described carefully checking AI output but still worried about becoming someone who only evaluates AI-generated answers rather than solving problems independently. This case shows that reflective collaboration can protect ownership in the immediate task while not fully eliminating concerns about future professional identity. Negative and boundary cases, therefore, refine the model: AI use becomes psychologically risky not simply when it is frequent, but when students lose control, understanding, self-investment, or opportunities to recalibrate anxiety through supportive feedback.

### 4.8 The psychological ownership–contextual support model

The final theme integrates the findings into the psychological ownership–contextual support model of GenAI-assisted learning. The model is best understood as a theory-generating conceptual map derived from qualitative thematic analysis. It does not suggest that universities should ban GenAI, nor does it claim that individual willpower alone can solve dependence. Instead, it proposes that reflective human–AI collaboration becomes more likely when learning environments make the learning process visible and psychologically consequential. The model’s value lies in organizing a set of testable relationships for future research rather than in providing statistical causal evidence in this study.

Four support levers emerged from the data. First, process-oriented assessment can require students to submit drafts, prompts, revisions, and reflective explanations. Second, dialogic feedback and oral defense can require students to explain and revise their work in conversation. Third, clear AI policy and disclosure can reduce ambiguity and make responsible use discussable. Fourth, student support and AI literacy can help students develop strategies for prompting, evaluating, revising, and taking ownership of AI-assisted work.

These levers are not merely administrative tools. They are psychological supports because they protect agency, self-regulation, and ownership. They help students move from dependent AI outsourcing toward reflective human–AI collaboration by making planning, evaluation, revision, and authorship necessary parts of the task. Table 3 maps these contextual support levers to the risk patterns and desired psychological outcomes identified in the model.

**Table 3:** Contextual support mapping in the psychological ownership–contextual support model.

Dimension	Risk pattern	Supportive condition	Desired psychological outcome
Assessment climate	Final-product grading encourages outsourcing	Staged submission, process logs, oral explanation	Greater ownership and accountability
Feedback climate	Limited feedback weakens monitoring and revision	Dialogic feedback and revision opportunities	Improved self-regulation
AI-policy clarity	Ambiguous rules encourage hidden use	Clear policy and AI-use disclosure	Responsible and visible AI use
Perceived educational support	Students feel judged or unsupported	Guidance, psychological safety, AI-literacy support	Lower competency anxiety
Reflective collaboration	AI replaces planning, judgment, or authorship	Plan, prompt, evaluate, revise, and own	Stronger ownership, recall, meaning, and confidence

## 5 Discussion

### 5.1 Psychological ownership in AI-assisted learning

The central psychological finding is that, in participants’ accounts, AI-assisted learning appeared to depend on whether the learner remained cognitively and authorially present. Dependent AI outsourcing was associated with weaker psychological ownership: students described having less control over the reasoning process, less intimate knowledge of the content, and less self-investment in the final product. Reflective collaboration was associated with retained ownership when students set goals, evaluated AI output, revised in their own voice, and could explain the final work.

This contributes to psychological ownership theory by extending it to human–AI collaboration. Although psychological ownership has previously been examined in educational and technology-use contexts, its application to students’ collaboration with generative systems remains underdeveloped, and the present accounts suggest one way to specify it. Ownership does not appear to require that every part of the work be produced without assistance. Rather, in participants’ accounts, ownership could still be experienced through reflective interaction with a non-human agent when the learner retained cognitive control, authorial responsibility, and a sense of personal investment. This qualifies the assumption that ownership requires purely unaided human effort. This framing also helps clarify what psychological ownership may add beyond adjacent constructs. Cognitive offloading helps explain why externalizing a task can reduce retention, and self-efficacy concerns whether students believe they can operate the tool; neither, on its own, explains why two students who complete equally polished, equally offloaded tasks can differ sharply in whether the work feels like it is theirs. Psychological ownership offers a way to interpret this residual difference by locating it in control, intimate knowledge, and self-investment in the reasoning process rather than in either memory load or tool confidence. It, thus, functions in this account as a process-level construct that links observable AI-use behavior to the affective and identity-related experiences—weak authorship, thin recall, and competency anxiety—that offloading and self-efficacy describe only in part.

### 5.2 Competency anxiety as a metacognitive signal

A second contribution is a more precise conceptualization of competency anxiety. The construct refers to a future-oriented, domain-specific concern that AI-supported performance may be outpacing the development or retention of an individual’s independently available capability. It is, therefore, an appraisal of a performance–capability discrepancy, not a general fear of technology or simply low AI self-efficacy.

Its psychological function is conditional. Competency anxiety may become an adaptive metacognitive signal when students can identify a specific skill gap, view it as controllable, and have supportive opportunities to practice, disclose, and revise their AI use. Under these conditions, anxiety can prompt recalibration and the recovery of ownership. By contrast, when the concern is interpreted as a global personal deficiency, when AI rules are ambiguous, or when disclosure feels unsafe, the same anxiety may produce shame, avoidance, or concealment. Thus, the manuscript distinguishes the construct itself from the regulatory pathway that follows it.

It remains possible for a student to have high AI-operation self-efficacy, such as knowing how to prompt and evaluate AI output, while experiencing high competency anxiety about domain-specific skills. Future measurement should therefore assess AI-operation self-efficacy and competency anxiety separately and should distinguish concern about writing, reasoning, coding, or professional judgment from discomfort with the tool itself.

### 5.3 Reflective collaboration and self-regulated learning

The findings also refine self-regulated learning in the context of GenAI. Reflective users enacted a cycle of planning, prompting, evaluating, revising, and owning. This cycle maps onto the forethought, performance, and self-reflection phases: students first formed their own intention, then monitored AI output during task performance, and finally revised and explained the work in relation to their own understanding.

Perceived educational support operated as a boundary condition for this cycle. Feedback, clear AI-use guidance, and psychologically safe discussion did not simply provide external resources; they helped students maintain metacognitive monitoring and authorial responsibility. When support was absent, students were more likely to interpret dependence as private failure and to hide AI use rather than transform it into reflective practice.

### 5.4 Cultural and institutional transferability: the Chinese higher-education context

The exclusive Chinese sample is theoretically consequential and should not be treated as a minor demographic limitation. In the sampled universities, dependent outsourcing was often narrated within a configuration of final-product assessment, limited formative feedback, large-class constraints, uncertain AI rules, and reluctance to discuss use openly when students anticipated judgment. Chinese university assessment research has similarly documented tensions between performance-oriented and learning-oriented assessment environments, as well as the difficulty of translating formative assessment principles into routine practice (Cheng et al., 2015; Wu et al., 2021). These conditions may amplify the attractiveness of polished AI output and weaken opportunities for students to reconstruct and defend their own reasoning.

At the same time, these features should not be essentialized as fixed properties of Chinese learners or universities. The five sites were heterogeneous, and reflective collaboration appeared wherever courses made drafts, dialog, explanation, and responsible AI use visible. The relevant cultural and institutional mechanism is, therefore, not nationality itself, but the interaction among the assessment authority, the perceived safety of negotiating expectations with instructors, feedback capacity, and the visibility of the process. In settings where students can openly question AI output and discuss acceptable use, competency anxiety may be more readily converted into recalibration rather than concealment.

The model’s transferability is consequently configurational rather than universal. Similar ownership losses may occur outside China wherever GenAI can generate acceptable products faster than learners can build understanding and where the assessment process remains invisible. Conversely, the pattern may be weaker within Chinese courses that use dialogic feedback and process evidence. Cross-national research should, therefore, compare matched task and assessment configurations rather than treating a country as a sufficient explanatory variable.

### 5.5 Task outsourcing affordance as a boundary condition

The disciplinary pattern observed in the data is best explained through task outsourcing affordance. Tasks are especially vulnerable when GenAI can generate the valued output, the learner’s process is not observable, and no independent explanation is required. By contrast, visible iteration, situated performance, source justification, debugging, and oral defense reduce the usefulness of outsourcing because the learner must retain intimate knowledge of the work.

Task outsourcing affordance is treated as an exogenous boundary condition rather than as a core stage in the psychological ownership pathway. It changes how easily dependent outsourcing can occur and how strongly an AI-use pattern is likely to affect ownership, but it is not itself a psychological mechanism. This is why it is incorporated into the propositions and contextual matrix rather than added as another central node in the conceptual map.

### 5.6 Future-testable propositions

Because this study is qualitative and theory-generating, these propositions are not tested here. To make them usable in subsequent research, each proposition is accompanied by indicative operationalization options rather than a prescriptive measurement model.

#### 5.6.1 Proposition 1

Greater engagement in dependent AI outsourcing (vs. reflective human–AI collaboration) will be associated with weaker psychological ownership even after accounting for prior achievement and the frequency of GenAI use. AI-use pattern could be operationalized with task-specific items and process indicators assessing whether students formed an initial plan, how much core reasoning or wording was generated before their own attempt, the extent of checking and revision, and whether they could justify accepted or rejected AI suggestions; prompt histories and revision logs could supplement self-report.

#### 5.6.2 Proposition 2

Psychological ownership will mediate the relationship between AI-use patterns and psychological learning outcomes. Task-specific ownership measures could adapt item wording from established psychological ownership scales (e.g., Avey et al., 2009) while separately assessing perceived control, intimate knowledge, and self-investment. Outcomes could combine delayed recall, oral-defense performance, explanation accuracy, perceived task meaningfulness, and a newly developed and validated competency-anxiety measure focused on the perceived discrepancy between AI-supported performance and independently available domain capability.

#### 5.6.3 Proposition 3

Perceived educational support will moderate the association between AI-use patterns and psychological ownership. Support could be measured through student perceptions of feedback usefulness, opportunities for revision, AI-policy clarity, psychological safety in disclosing uncertainty or AI use, and access to AI-literacy guidance; course-level indicators such as staged submission, oral defense, and disclosure requirements could be modeled alongside individual perceptions.

#### 5.6.4 Proposition 4

Task outsourcing affordance will moderate the relationship between AI-use pattern and psychological ownership, such that dependent outsourcing will be more strongly associated with ownership loss when outputs are readily generatable, process evidence is absent, and explanation is not required. Future studies could code task affordance using generability of the final product, visibility of the production process, requirement for independent explanation, and traceability of authorship, and could experimentally vary these features within the same discipline.

### 5.7 Practical implications

Practical implications follow from this account. From a psychological perspective, task designs that require students to plan, explain, and revise are likely to preserve the three ownership pathways—control, intimate knowledge, and self-investment—even when AI is used. For example, an AI-assisted writing task could require an initial outline written before AI use, a brief AI-use log, a revised draft, and an explanation of which suggestions were accepted, rejected, or rewritten. These recommendations are consistent with recent guidance that emphasizes assessment redesign, transparent expectations for AI use, and proportionate support for teachers (Khlaif et al., 2024; Miao and Holmes, 2023; Moorhouse et al., 2023). However, the faculty strand shows that these designs must be proportionate to class size and workload. Sampling a subset of oral defenses, using structured peer feedback, short process annotations, or staged checkpoints may preserve process visibility without requiring intensive individual feedback on every submission.

Student support should also address competency anxiety without stigmatizing students as dishonest or weak. AI-literacy support is useful, but it should be paired with opportunities to practice domain-specific reasoning, writing, coding, or problem-solving so that students rebuild confidence in their own abilities while learning to collaborate with AI responsibly (Bittle and El-Gayar, 2025; Johnston et al., 2024; Kelly et al., 2023; Mai et al., 2024). Examples of such AI-irreplaceable abilities include formulating novel research questions, evaluating the credibility of sources beyond surface plausibility, and making ethical judgments about AI-generated content.

### 5.8 Limitations and future research

This study has limitations. First, it was conducted across five universities within a single Chinese higher-education context. The discussion identifies ways in which product-oriented assessment, feedback constraints, instructor authority, policy ambiguity, and the perceived safety of disclosure may have shaped the observed dynamics. However, this study cannot determine which features are culturally specific and which reflect more general assessment configurations. The findings should not be generalized to Chinese higher education as a whole, and cross-national comparisons should match task type, class size, feedback design, and AI-policy conditions rather than using country as a proxy for culture. Future research should include multinational comparative studies, including samples from Latin America, Europe, and South Asia, to examine whether similar ownership and competency-anxiety dynamics appear under different educational cultures and institutional policy environments. Second, discipline patterns were interpreted through task outsourcing affordance, but the sample was not homogeneous across STEM, humanities/social sciences, and arts-related fields. Future work should balance disciplinary distribution where possible and should systematically sample matched task types within each discipline. This would allow researchers to test whether generatability, process visibility, and explanation requirements predict ownership more strongly than broad disciplinary labels. Third, this study is an exploratory qualitative interview-based study supported by supplementary background information. The contrasts reported in the findings are interpretive summaries rather than scale scores or inferential statistical tests, and the propositions require quantitative, longitudinal, or experimental testing.

Fourth, this study relies on self-reported experience and participant-compiled critical incident records. Although these sources are appropriate for examining lived psychological experience, they may be affected by recall bias, social desirability, and uneven detail across participants. Future research should attempt methodological triangulation by combining interviews with qualitative and quantitative instruments, such as behavioral tasks, writing-process data, prompt and revision logs, learning analytics, validated survey scales, rubric-based oral-defense performance, or longitudinal tracking. Fifth, this study identifies learning-environment conditions associated with reflective use but does not experimentally test interventions. Future work could evaluate whether process-oriented assessment, AI-use reflection, or feedback redesign actually reduces dependent outsourcing and strengthens psychological ownership.

Sixth, this study reflects students’ experiences with GenAI at a specific stage of technological development. As GenAI systems become more multimodal, embedded in learning platforms, and capable of supporting speech, design, coding, laboratory simulation, and discipline-specific workflows, the mechanisms of psychological ownership and competency anxiety may shift. Future studies should examine whether the distinction between dependent outsourcing and reflective collaboration remains stable across more embedded and multimodal AI environments.

Seventh, this study identified assessment and feedback conditions that may support reflective AI use, but it did not examine the institutional costs of implementing such support. Process-oriented assessment, dialogic feedback, and AI-literacy guidance require time, staffing, workload recognition, and curriculum-level coordination. Future research should examine scalable designs and the institutional investment needed to make such support feasible in large-class and resource-constrained contexts.

Finally, this study does not measure objective learning gains or long-term competency development. The findings concern psychological experience as a necessary but not sufficient condition for learning. Future research should link ownership and anxiety to behavioral measures of retention, transfer, independent problem-solving, or performance under timed conditions.

## 6 Conclusion

This study examined how university students at five Chinese universities experienced GenAI-assisted academic work and how learning environments and task affordances shaped movement from dependent AI outsourcing toward reflective human–AI collaboration. The findings suggest that the most important question is not whether students use AI, but whether they remain cognitively and authorially present in the learning process. When GenAI replaces planning, reasoning, and revision, students may submit polished work while experiencing weak psychological ownership, shallow memory and explainability, reduced meaning-making, and heightened competency anxiety. When students use GenAI reflectively, they can receive substantial support while retaining agency, authorship, and responsibility.

This study proposes the psychological ownership–contextual support model of GenAI-assisted learning as a theory-generating, context-sensitive conceptual map. Assessment climate, feedback practices, AI-policy clarity, perceived educational support, and task outsourcing affordance may make either dependence or collaboration more likely. Faculty and administrator accounts further show that ownership-preserving designs must be institutionally feasible and supported by a coherent curriculum and policy. The implication is not that institutions should detect AI use more aggressively, but that learning environments should require and support planning, explaining, revising, and owning work. In the AI era, the goal of learning is not to keep students away from intelligent tools, but to help them use those tools without losing their own thinking.

## Data Availability

The raw data supporting the conclusions of this article will be made available by the authors, without undue reservation.
