# Supplementary material for: From AI dependence to reflective collaboration: psychological ownership, competency anxiety, and perceived support in AI-assisted learning
Source: Front Psychol. 2026 Jul 15;17:1903784. doi: 10.3389/fpsyg.2026.1903784 (PMC13416268; doi:10.3389/fpsyg.2026.1903784)
Supplement: Supplementary file 1 [file Supplementary_file_1.DOCX]

**Supplementary Material**

# Supplementary Table S1. Coding framework

| **First-order code** | **Second-order manifestations** | **Theoretical anchor** |
| --- | --- | --- |
| Psychological ownership | Sense that work is mine/not mine; perceived control; intimate knowledge; self-investment; authorship; responsibility for arguments | Psychological ownership pathways |
| Memory and explainability | Ability to recall structure, explain reasoning, defend choices, remember learning process | Cognitive offloading; retrieval and learning |
| Meaning-making | Personal voice, value judgment, emotional connection, perceived meaningfulness of task | Self-determination theory; meaning in learning |
| Self-regulated AI use | Thinking before prompting, monitoring GenAI output, evaluating reliability, revising in own voice | Self-regulated learning; metacognition |
| Dependent AI outsourcing | Direct generation of main structure/content; little checking; accepting output; difficulty explaining work | Cognitive offloading; dependency risk |
| Assessment climate | Final-product grading, process documentation, oral defense, draft submission, rubric orientation | Learning environment climate; assessment design |
| Feedback climate | Timeliness, dialogic feedback, teacher comments, peer feedback, revision opportunities | Feedback literacy; learning climate |
| AI-policy clarity | Rules on acceptable AI use, disclosure requirements, enforcement, perceived fairness | Policy clarity (learning context) |
| Perceived educational support | Teacher guidance, advising, AI literacy resources, non-punitive support, institutional care | Educational support; teacher and institutional support |
| Competency anxiety | Future-oriented, domain-specific concern that AI-supported performance exceeds independently available capability; perceived performance-capability discrepancy; worries about employability, writing, coding, reasoning, or problem-solving decline | Future self-concept; domain-specific self-efficacy; metacognitive appraisal |
| Institutional learning context | Resource differences, class size, formative assessment capacity, guidance availability | Institutional context (exploratory) |
| Task outsourcing affordance | Generatability of final output; process visibility; requirement for explanation or defense; traceability of authorship; iterative or embodied production | Task affordance; assessment design boundary condition |
| Teacher and administrator responses | Curriculum redesign, feedback methods, AI-use declarations, support strategies | Instructional or support intervention |
| Competency-anxiety response pattern | Adaptive recalibration (practice, reduced outsourcing, checking) versus defensive response (shame, concealment, avoidance); coded separately from anxiety itself | Metacognitive regulation; psychological safety; perceived controllability |
| Faculty/administrator analytic strand | Definitions of problematic dependence; ownership and explainability markers; assessment feasibility; workload; AI-policy ambiguity; curriculum responsibility; support priorities | Stakeholder analysis; institutional implementation context |

# Supplementary Table S2. Student participant profile

| **ID** | **Gender** | **Age** | **Level** | **Site** | **Discipline** | **AI freq.** |
| --- | --- | --- | --- | --- | --- | --- |
| A01 | F | 20-22 | UG | Site A | HSS | 5-7/wk |
| A02 | M | 20-22 | UG | Site A | HSS | 4-6/wk |
| A03 | F | 20-22 | UG | Site A | HSS | 3-4/wk |
| A04 | F | 20-22 | UG | Site A | HSS | 6-8/wk |
| A05 | M | 20-22 | UG | Site A | STEM | 7-10/wk |
| A06 | F | 20-22 | UG | Site A | STEM | 5-7/wk |
| A07 | M | 23-25 | PG | Site A | STEM | 8-10/wk |
| A08 | F | 23-25 | PG | Site A | HSS | 4-5/wk |
| A09 | M | 20-22 | UG | Site A | Arts | 1-2/wk |
| A10 | F | 23-25 | PG | Site A | Arts | 2-3/wk |
| B01 | M | 20-22 | UG | Site B | STEM | 6-8/wk |
| B02 | F | 20-22 | UG | Site B | STEM | 5-6/wk |
| B03 | M | 23-25 | PG | Site B | STEM | 7-9/wk |
| B04 | F | 20-22 | UG | Site B | STEM | 8-10/wk |
| B05 | M | 20-22 | UG | Site B | STEM | 4-5/wk |
| B06 | F | 20-22 | UG | Site B | HSS | 3-4/wk |
| B07 | M | 23-25 | PG | Site B | STEM | 10+/wk |
| B08 | F | 20-22 | UG | Site B | Arts | 2-3/wk |
| B09 | M | 20-22 | UG | Site B | STEM | 3-4/wk |
| B10 | F | 23-25 | PG | Site B | HSS | 4-5/wk |
| C01 | F | 20-22 | UG | Site C | HSS | 3-4/wk |
| C02 | M | 20-22 | UG | Site C | HSS | 4-5/wk |
| C03 | F | 20-22 | UG | Site C | HSS | 5-6/wk |
| C04 | M | 20-22 | UG | Site C | STEM | 3-4/wk |
| C05 | F | 23-25 | PG | Site C | HSS | 3-4/wk |
| C06 | F | 20-22 | UG | Site C | Arts | 1-2/wk |
| C07 | M | 20-22 | UG | Site C | STEM | 4-5/wk |
| C08 | F | 23-25 | PG | Site C | Arts | 2-3/wk |
| C09 | M | 20-22 | UG | Site C | HSS | 5-6/wk |
| C10 | F | 20-22 | UG | Site C | STEM | 3-4/wk |
| D01 | F | 20-22 | UG | Site D | HSS | 6-8/wk |
| D02 | M | 20-22 | UG | Site D | HSS | 5-7/wk |
| D03 | F | 23-25 | UG | Site D | HSS | 4-5/wk |
| D04 | M | 20-22 | UG | Site D | STEM | 7-9/wk |
| D05 | F | 20-22 | UG | Site D | STEM | 5-6/wk |
| D06 | F | 20-22 | UG | Site D | HSS | 6-7/wk |
| D07 | M | 23-25 | PG | Site D | STEM | 8-10/wk |
| D08 | F | 20-22 | UG | Site D | Arts | 2-3/wk |
| D09 | M | 23-25 | PG | Site D | HSS | 4-5/wk |
| D10 | F | 23-25 | PG | Site D | Arts | 3-4/wk |
| E01 | M | 20-22 | UG | Site E | STEM | 6-8/wk |
| E02 | F | 20-22 | UG | Site E | STEM | 5-6/wk |
| E03 | M | 20-22 | UG | Site E | STEM | 4-5/wk |
| E04 | F | 20-22 | UG | Site E | STEM | 3-4/wk |
| E05 | M | 23-25 | PG | Site E | STEM | 7-9/wk |
| E06 | F | 20-22 | UG | Site E | HSS | 5-6/wk |
| E07 | M | 20-22 | UG | Site E | HSS | 4-5/wk |
| E08 | F | 23-25 | PG | Site E | STEM | 6-7/wk |
| E09 | M | 20-22 | UG | Site E | Arts | 1-2/wk |
| E10 | F | 23-25 | PG | Site E | Arts | 3-4/wk |

Note. The student profile is intentionally reported with broad categories only. HSS = Humanities and Social Sciences. Site A–E are anonymized data-source codes and do not represent institutional hierarchy, ranking, or quality. Consistent with the sampling described in the main text, the sites span varied institutional contexts to support contextual diversity; however, this information was used only for sample description and not for subgroup comparison. Specific majors, exact ages, and institution-type labels are not reported to reduce re-identification risk.

# Supplementary Table S3. Faculty and administrator participant profile

| **ID** | **Role category** | **Broad area** | **Experience range** |
| --- | --- | --- | --- |
| T01 | Professor | Education | 16+ years |
| T02 | Associate Professor | Humanities | 11-15 years |
| T03 | Associate Professor | STEM | 6-10 years |
| T04 | Associate Professor | STEM | 6-10 years |
| T05 | Professor | STEM | 16+ years |
| T06 | Lecturer | Humanities | 0-5 years |
| T07 | Professor | Design | 11-15 years |
| T08 | Lecturer | Humanities | 0-5 years |
| T09 | Associate Professor | STEM | 6-10 years |
| T10 | Administrator | Academic affairs / administration | 6-10 years |

Note. Faculty and administrator information is reported in broad categories only to preserve anonymity.

# Supplementary Table S4. Trustworthiness measures in the qualitative design

| **Quality dimension** | **Specific measures** |
| --- | --- |
| Credibility | Member checking with 10 students and 3 faculty/administrator participants; sustained engagement; triangulation across student interviews, faculty interviews, critical incident records, and background information forms; separate learner and stakeholder analytic strands followed by convergence/divergence comparison. |
| Transferability | Thick description of national and institutional context, discipline, academic level, task outsourcing affordance, assessment climate, feedback climate, and AI-policy context; explicit non-essentializing treatment of the Chinese sample; detailed sampling matrix in Supplementary Tables S2 and S3. |
| Dependability | NVivo coding logs, analytical memos, strand-specific matrices, and independent coding records; double coding of 10 student interview transcripts (20%) and 3 faculty/administrator interview transcripts (30%); inter-coder reliability checks (kappa = 0.82 and 0.79); bi-weekly coding meetings. |
| Confirmability | Researcher reflexivity journals; external expert review of the coding framework; traceability from original excerpts to psychological and contextual codes; anonymized examples available upon reasonable request. |

# Supplementary Methods 1. Ethics, consent, anonymization, and data-protection summary

The study was conducted in accordance with accepted ethical standards for social science and educational research involving human participants. Ethical approval was obtained before data collection, and all participants provided written informed consent before taking part in the study.

Participants were informed of the study purpose, the voluntary nature of participation, their right to withdraw, and the anonymization procedures. They were also informed that no individual AI-use behavior would be reported to teachers or administrators for disciplinary purposes.

To protect confidentiality, names of persons, universities, courses, and other potentially identifying details were removed or replaced with participant codes and broad contextual categories. Student sites were reported as Site A–E only. Exact ages, specific majors, and institution-type labels were not publicly reported.

Audio recordings, transcripts, background information forms, and critical incident records were stored in de-identified form and used only for academic research. Complete transcripts and full critical incident records are not publicly available because they contain contextual details that could lead to participant or institutional identification, and because participants did not consent to public release of their full records.

# Supplementary Methods 2. Student in-depth semi-structured interview protocol

## Module A. Background and GenAI use context

- Could you briefly introduce your academic background and typical learning tasks?

- Which GenAI tools have you used for academic work during the past semester?

- How often do you use GenAI and for what types of tasks?

## Module B. Psychological ownership, memory, and meaning

- After completing a GenAI-assisted assignment, does the final work feel like yours? Why or why not?

- Can you usually recall the main argument, structure, or reasoning after using GenAI?

- Have you ever felt that a GenAI-assisted product was polished but emotionally empty or disconnected from you?

## Module C. Self-regulated versus dependent AI outsourcing

- What do you usually do before asking AI for help?

- Do you evaluate, challenge, or revise GenAI output? Please give an example.

- Have you ever used AI as a substitute for thinking? What happened?

## Module D. Assessment and feedback climate

- What types of assignments are common in your program?

- Are drafts, process documents, oral explanations, or reflections required?

- How does teacher feedback influence your decision to use GenAI?

## Module E. AI-policy clarity and perceived educational support

- Does your university or department provide clear guidance on acceptable AI use?

- Do you feel supported or mainly monitored when using GenAI for learning?

- What kinds of teacher or institutional support would help you use GenAI more reflectively?

## Module F. Competency anxiety and future identity

- When AI can complete many academic tasks, which abilities do you worry may decline?

- What AI-irreplaceable abilities do you think students need to develop?

- Have you felt anxiety about future employment or professional competence because of AI?

## Module G. Closing reflection

- What is the most important effect of AI on your learning experience?

- What advice would you give to teachers or student-support staff?

# Supplementary Methods 3. Faculty/administrator semi-structured interview protocol

## Module A. Professional background

- Could you briefly describe your teaching, advising, or administrative role?

- What student groups or programs are you most familiar with?

## Module B. Observations of student AI use

- What changes have you observed in students' academic work since GenAI became widely used?

- Have you observed polished work that students could not explain?

- Which tasks most often invite direct AI outsourcing?

## Module C. Assessment and feedback climate

- How are assignments usually assessed in your program or courses?

- To what extent are drafts, process documentation, oral defense, reflective logs, or formative feedback used?

- What constraints make formative assessment difficult?

## Module D. AI-policy clarity and perceived educational support

- Does your institution or department have AI-use policies or declaration requirements?

- How clear and enforceable are these policies in practice?

- What support do teachers need to guide students effectively?

## Module E. Student support and competency anxiety

- Have you noticed students experiencing anxiety or loss of confidence because of AI?

- What warning signs indicate problematic AI dependence?

- What advising, curriculum, or student-affairs interventions are feasible?

## Module F. Closing reflection

- What is the most urgent contextual challenge GenAI creates for higher education?

- What should universities do first?

# Supplementary Table S5. Background information form and screening items

| **Item** | **Prompt** | **Response options / notes** |
| --- | --- | --- |
| 1 | Participant code | Completed by researcher; no names |
| 2 | Age range | 20-22 / 23-25 / other broad range if needed |
| 3 | Gender | Female / Male / Non-binary / Prefer not to say / Other |
| 4 | Academic level | Undergraduate / Postgraduate |
| 5 | Broad discipline | STEM / Humanities and Social Sciences / Arts / Other |
| 6 | Frequency of GenAI use for academic tasks | Categorical background item used for sampling awareness only |
| 7 | Main academic tasks supported by AI | Literature search / summarizing / essay drafting / polishing / coding / translation / idea generation / other |
| 8 | Recent AI-assisted learning episode | Participant indicates whether they are willing to discuss one concrete episode in the interview |
| Note | Use of the form | The form collected minimal background information only. It was used for sample description and interview preparation, not for scale scoring, covariate analysis, or statistical comparison. |

# Supplementary Table S6. Student critical incident record template

| **Field** | **Response** |
| --- | --- |
| Participant code |  |
| Date of incident |  |
| Type of academic task | Essay, report, coding task, literature review, presentation, translation, design work, etc. |
| AI tool used | Optional; generic names are acceptable |
| What I asked AI to do | Briefly describe the prompt or purpose; do not paste private content |
| What AI produced | Brief description of output type, not full text |
| What I did myself | Thinking, editing, checking, decision-making, reflection, or revision contributed by the student |
| Sense of psychological ownership | Did the final work feel like yours? Why or why not? |
| Memory and explainability | After completion, could you recall and explain the core content? |
| Self-regulation | Did you plan, monitor, evaluate, or revise GenAI output? |
| Emotional experience | Satisfied / empty / anxious / confident / confused / other; explain briefly |
| Competency reflection | Did this incident strengthen or weaken your confidence in your own ability? |
| Learning outcome | What did you learn, if anything? |
| Reflection on AI use | Did AI help, replace, or weaken your thinking in this incident? |

# Supplementary Table S7. Task-outsourcing-affordance contextual matrix (non-inferential)

| **Task context** | **Task-affordance profile** | **Ownership-related vulnerability** | **Interpretation for support design** |
| --- | --- | --- | --- |
| AI-generatable essays or reports | High generatability; low process visibility; standardized final product; explanation often not required | Students may accept AI-produced structure and wording without developing personal interpretation or authorial history | Use staged drafts, source justification, reflective explanation, and feedback on argument development |
| Coding or problem-solving tasks | Affordance varies: high when only functioning output is rewarded; lower when debugging and explanation are assessed | Students may submit functioning code or solutions that they cannot reconstruct, diagnose, or defend | Require code walkthroughs, error diagnosis, design-choice explanation, and selective prompt-use reflection |
| Seminar, project, or research-training settings | Lower affordance when discussion, revision, source evaluation, and process evidence are visible | Frequent AI use can remain compatible with ownership when students plan, evaluate, and revise | Preserve reflective cycles of plan, prompt, evaluate, revise, explain, and own |
| Studio, design, or performance-based tasks | Often lower affordance because iteration, embodied production, and visible authorship are built into the task | AI may support ideation but can blur originality or displace judgment when contribution boundaries are unclear | Clarify acceptable AI reference use and require documentation of personal decisions and iterative contribution |

Note. This matrix summarizes task-level boundary conditions identified in the qualitative analysis. It is not a scale, ranking, statistical subgroup comparison, or claim that any discipline is inherently more vulnerable to AI outsourcing.

# Supplementary Table S8. Reported interview and critical-incident excerpt consistency check

| **Manuscript location** | **Code / record ID** | **Source type** | **Confirmed de-identified profile or incident category** | **Consistency check outcome** |
| --- | --- | --- | --- | --- |
| Section 4.1: inability to explain AI-assisted essay | C02 | Student interview | Site C; male; undergraduate; HSS; age 20-22; GenAI use 4-5 times/week | Matches the HSS writing/explainability episode reported in the results. |
| Section 4.2: feedback vs. final-grade-only courses | D02 | Student interview | Site D; male; undergraduate; HSS; age 20-22; GenAI use 5-7 times/week | Matches the assessment-climate and feedback-deprivation theme. |
| Section 4.4: algorithm-first reflective routine | C04 | Student interview | Site C; male; undergraduate; STEM; age 20-22; GenAI use 3-4 times/week | Matches the STEM algorithm/coding episode and reflective collaboration theme. |
| Section 4.5: coding-interview competency anxiety | B04 | Student interview | Site B; female; undergraduate; STEM; age 20-22; GenAI use 8-10 times/week | Matches the STEM competency-anxiety episode reported in the results. |
| Section 4.7: high-frequency AI user retaining ownership | B07 | Student interview | Site B; male; postgraduate; STEM; age 23-25; GenAI use 10+ times/week | Matches the postgraduate STEM high-frequency use and boundary-case analysis. |
| Section 4.1: lab-report discussion section | CIR-023 | Critical incident record | Lab report / discussion-writing episode; dependent AI outsourcing; difficulty answering a TA question | Matches the loss-of-ownership, memory, and explainability theme. |
| Section 4.1: generated code passed tests but could not be explained | CIR-047 | Critical incident record | Coding task; AI-generated code; difficulty explaining loop logic in viva | Matches the dependent outsourcing and explainability-risk theme. |
| Section 4.4: AI-suggested counterarguments after student outline | CIR-076 | Critical incident record | Essay planning and counterargument episode; student planned, evaluated, rejected/revised AI output | Matches the reflective human-AI collaboration theme. |
| Section 4.4: code-comment polishing after line-by-line understanding | CIR-091 | Critical incident record | Coding/comment-polishing episode; student retained line-by-line understanding before using AI | Matches the self-regulated AI-use and ownership-retention theme. |
| Section 4.5: AI-assisted literature review with exam-condition concern | CIR-112 | Critical incident record | Literature review episode; performance-competence mismatch; anxiety about independent writing | Matches the competency-anxiety theme. |
| Section 4.5: becoming an AI supervisor rather than a learner | CIR-128 | Critical incident record | Problem-solving/coding-related episode; concern about ability if AI access disappeared | Matches the future-oriented competency-anxiety theme. |

Note. This table documents consistency between reported excerpts, participant codes/CIR IDs, and anonymized profiles or incident categories. Raw Chinese transcripts and full critical incident records are not reproduced publicly for confidentiality reasons.

# Supplementary Table S9. Faculty/administrator analytic-strand summary

| **Analytic issue** | **Distinct faculty/administrator perspective** | **Relation to student accounts** | **Illustrative evidence reported in main text** |
| --- | --- | --- | --- |
| Evaluative judgment and problematic dependence | Problematic use was framed through inability to judge, explain, or take responsibility for AI-assisted work rather than frequency alone. | Converges with students who retained ownership despite frequent AI use when they could explain and overrule AI. | T01: students must learn "how to judge AI, not only how to use AI" (Section 4.6). |
| Assessment feasibility and workload | Process-oriented assessment and formative feedback were viewed as desirable but difficult to sustain in large classes and under workload constraints. | Complicates student interpretations that limited feedback simply signals that only the final product matters. | T08: formative feedback is difficult with 80 students in a class (Section 4.6). |
| Curriculum and policy coordination | AI literacy, disclosure expectations, and independently demonstrable capabilities were viewed as curriculum-level responsibilities, not only individual teacher choices. | Extends student accounts of unclear rules and perceived lack of support to policy and implementation capacity. | T10: curriculum design had not caught up with competencies required in an AI-rich environment (Section 4.6). |
| Stakeholder divergence | Institutional silence was described as partly arising from uncertainty, training gaps, and implementation capacity. | Students often experienced the same silence as judgment, indifference, or lack of psychological safety. | Cross-strand analytic synthesis; no statistical comparison. |

Note. This table summarizes the distinct analytic contribution of the faculty and administrator interviews. It is an interpretive matrix, not a frequency table or statistical comparison.
